# Pulmonary microbial spectrum of *Burkholderia multivorans* infection identified by metagenomic sequencing

**DOI:** 10.3389/fmed.2025.1577363

**Published:** 2025-06-17

**Authors:** Hui Xu, Ruixue Zhang, Xiaoxue Zhang, Zhi Zhang, Yingmei Feng, Lianjun Lin

**Affiliations:** ^1^Department of Geriatrics, Peking University First Hospital, Beijing, China; ^2^National Engineering Research Center for Beijing Biochip Technology, CapitalBio Corporation, Beijing, China; ^3^Department of Science and Technology, Beijing Youan Hospital, Capital Medical University, Beijing, China

**Keywords:** *B. multivorans*, infection, metagenomic sequencing, antibiotic resistance, Burkholderia cepacia complex

## Abstract

**Purpose:**

*Burkholderia multivorans*, a Gram-negative bacterium, often infect patients with severe immunocompromised and cystic fibrosis. *B. multivorans* infection is challenging to treat due to its ability to disrupt the action of multiple antimicrobial agents through intrinsic and acquired resistance mechanisms. A better understanding of the pulmonary microbial spectrum of *B. multivorans* infection is crucial for the prevention and treatment of *B. multivorans*.

**Case presentation:**

This case series reviewed the respiratory microbiome structure and alternations during the treatment of *B. multivorans* infection through metagenomic next-generation sequencing (mNGS). Analysis of mNGS data of 19 pharyngeal secretion samples collected from the 3 COVID-19 patients at different time points showed that the relative abundance of *B. multivorans* was fluctuated and eventually increased, indicating the possible development of drug resistance. A total of 40 antibiotic-resistant genes (ARGs) were detected. Significantly, the levels of CEOA, CEOB, and OPCM were consistent with the trends in the relative abundance of *B. multivorans*. Besides, we described nine previously uncharacterized non-synonymous mutations in *PenA* of *B. multivorans*. These mutations lead to amino acid changes Thr32Ala, Ala43Ser, Gln105Arg, Asn202Ser, Gln219Arg, Gly241Ala, Val259Ala, Thr279Ala, and Ser298Ile that may associate with resistance to β-lactam antibiotics.

**Conclusion:**

This report shed light on the importance of rapidly diagnosis and treatment of *B. multivorans* infection. mNGS serve as a powerful microbial detection tool that provides a comprehensive, sensitive, and rapid method for pathogen detection and drug resistance analysis.

## Introduction

*Burkholderia multivorans*, a member of the Burkholderia cepacia complex (BCC), is an opportunistic pathogen, responsible for severe infections in cystic fibrosis and immunocompromised individuals (1). *B. multivorans* is associated with “cepacia syndrome”, a rapidly progressive necrotizing pneumonia (2). Infections caused by *B. multivorans* are often difficult to treat as this pathogen has a large genome (three chromosomes of ∼7.0 Mbp) that carries multiple antibiotic resistance genes, and is inherently resistant to a multitude of antibiotics. A major antibiotic resistance determinant of *B. multivorans* is an inducible class A β-lactamase of the Pen family, PenA. It possesses a very wide range of substrates, including carbapenems and β-lactamase inhibitors (3). Trimethoprim-sulfamethoxazole, minocycline, doxycycline, doripenem, meropenem, and ceftazidime have recommended as preferred therapies for BCC infections (4). However, resistance to ceftazidime can develop rapidly during treatment of infections caused by *B. multivorans.* Besides, PenA has a free-moving loop at the entrance to the active site, called the Ω-loop (5). It is a motif composed of sixteen amino acids (residues 164 to 179). The Ω-loop of PenA emerges as a “hot spot” for obtaining single amino acid substitution that extends the substrate heterogeneity of PenA in clinically isolated *B. multivorans* (3). Substitutions at residues 164, 167, 169, and 179 of PenA were often observed in clinical isolates, conferring resistance to ceftazidime (5).

COVID-19 is a global pandemic caused by severe acute respiratory syndrome coronavirus 2 (SARS-CoV-2). It has been reported that bacteria and fungi may cause co-infection in critically ill COVID-19 patients, elevating its morbidity and mortality (6). Despite previous studies reporting on the epidemiology and antibiotic resistance profiles of *B. multivorans* infection (7), the rapid diagnosis and treatment of *B. multivorans* infection in severe COVID-19 patients are not well-defined. The present study interpreted the results of metagenomic sequencing to determine the respiratory microbiome structure and alternations in 3 COVID-19 patients infected by *B. multivorans*. Importantly, we described nine previously uncharacterized non-synonymous mutations in PenA of *B. multivorans* that may associated with resistance to β-lactam antibiotics.

## Materials and methods

Three patients with COVID-19 infected by *B.multivorans* who were hospitalized between February and April 2020 were included. Demographic, laboratory, radiological data and therapeutic management were obtained from the medical records. Pharyngeal secretion samples were collected at different time points during hospitalization according to standard procedures (8) and delivered to the laboratory for metagenomic next-generation sequencing (mNGS) detection within 24 h. DNA was extracted using the MAPMI sample preparation Kit (CapitalBio Corporation, Beijing, China). Sequencing libraries were constructed through enzymatic DNA fragmentation (200–300bp), end repair, adapters ligation, and polymerase chain reaction amplification. Sequencing was performed using the BioelectronSeq 4000 sequencer (CapitalBio, Beijing, China). Quality control was carried out on the original sequencing data, and reads with a length of less than 50 bp, low quality and low complexity were removed. The remaining sequencing data were mapped to the human reference genome GRCh38 to remove the interference of the host sequence by Bowtie2 software (9). The microorganism composition of the samples was annotated, the microbial genomic sequences were assembled using SPAdes, and the abundance of antibiotic-resistant genes (ARGs) was analyzed in combination with the comparison results and the ARGs annotation database. Changes in the pulmonary microbiota and related drug-resistant genes during treatment were analyzed in combination with clinical medication information of the patients. The schedule of pharyngeal secretion samples collection from the same patients at different time points was indicated in Supplementary Table 1. A total of nineteen pharyngeal secretion samples were collected from the three patients. Besides, the antibiotic medications of the patient during hospitalization were summarized in Supplementary Table 2.

Study protocols were reviewed and approved by the Ethics Committee of Peking University First Hospital (approval No. 2020-258), Beijing, China. All procedures followed were in accordance with the ethical standards of the responsible committees and with Declaration of Helsinki. Written informed consent for the publication of case-related details was obtained from each participant or legal guardians.

## Results

**FIGURE 1:**
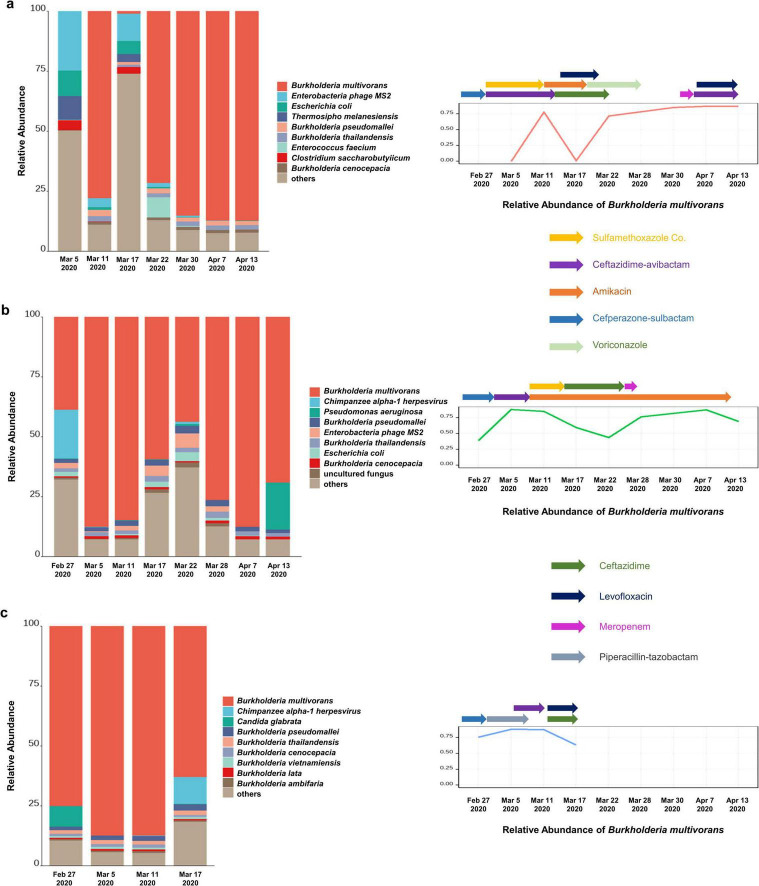
The abundance of *B. multivorans* in infected patients. **(a)** The relative abundance of *B. multivorans* during the treatment by metagenomic sequencing of patient 1; **(b)** the relative abundance of *B. multivorans* during the treatment by metagenomic sequencing of patient 2; **(c)** the relative abundance of *B. multivorans* during the treatment by metagenomic sequencing of patient 3.

Their age ranged from 59 to 77 years and two of them were male. Analysis of the mNGS data of nineteen pharyngeal secretion samples collected from three severe COVID-19 patients at different time points showed a higher relative abundance of *B. multivorans*. Besides, the relative abundance of *B. pseudomallei*, *B. thailandensis*, and *B. cenocepacia* of the same genus was also ranked high across different samples (Figure 1).

Patient 1, a 59-year-old man, was admitted for COVID-19 on Feb 4 2020. On the sixth day after hospitalization, he was transferred to the intensive care unit due to respiratory failure and received mechanical ventilation. PCR testing for SARS-CoV-2 became negative on day 17 of admission. Nevertheless, his condition deteriorated tenaciously. Data on metagenomic next-generation sequencing (mNGS) of pharyngeal secretion collected at post-sulfamethoxazole Co. and ceftazidime-avibactam treatment time point showed an increased relative abundance of *B. multivorans*. Subsequently, the therapeutic regimen was adjusted to amikacin and levofloxacin maintained for 1 week, and ceftazidime maintained for 2 weeks. Sputum culture examination became positive for BCC on march 30, at which time his therapy was switched to levofloxacin and piperacillin-tazobactam according to the results of antimicrobial susceptibility testing of the isolated BCC. There was a fluctuated relative abundance of *B. multivorans* and eventually increased (Figure 1a). Besides, the changes of inflammatory markers (white blood cell count, neutrophil count, and C-reactive protein) and procalcitonin of the patient during hospitalization were shown in Supplementary Figure 1. On May 18, the patient was discharged.

Patient 2, a 74-year-old woman with a history of hypertension, was admitted to the hospital due to fever, shortness of breath, and respiratory failure. Nasopharyngeal swab PCR tested positive for SARS-CoV-2 and the chest CT showed diffuse lesions in both lungs. The patient was subsequently diagnosed with severe COVID-19. On the third day after admission, she underwent endotracheal intubation and mechanical ventilatory support due to acute respiratory distress syndrome. PCR testing for SARS-CoV-2 became negative after 15 days of hospitalization. On Feb 27, pharyngeal secretion mNGS revealed *B. multivorans*. One day later, sputum culture was positive for BCC. Subsequently, the patient was successively treated with cefoperazone-sulbactam, ceftazidime-averbactam, amikacin, sulfamethoxazole Co., and ceftazidime (Supplementary Table 2). Supplementary Figure 2 presented the changes of inflammatory markers and procalcitonin. Nevertheless, data on pharyngeal secretion mNGS at different time points showed fluctuated relative abundance of *B. multivorans* (Figure 1b). Despite broadening antimicrobials, her condition exacerbation and succumbed by severe sepsis on day 78 of admission.

Patient 3, a 77-year-old man, presented with intermittent fever and oxygen saturation of 93%. Throat swab PCR tested positive for SARS-CoV-2. The patient was transferred to intensive care unit due to lung function deterioration and respiratory failure. Sputum samples collected post-meropenem, vancomycin, and caspofungin treatment exhibited culture-positive for BCC. Pharyngeal secretion mNGS test showed *B. multivorans*, at which time his therapy was switched to piperacillin-tazobactam and maintained for 1 week. Drug susceptibility testing on Mar 7 revealed that clinically isolated BCC was sensitive to ceftazidime (MIC: 2 ug/mL) and ciprofloxacin (MIC: ≤0.25 ug/mL), and was resistant to ampicillin, ampicillin-sulbactam, cefazolin, and imipenem. Ceftazidime-averbactam was administered between the second and third mNGS tests. Then, his therapeutic regimen was switched to ceftazidime and levofloxacin according to the results of pharyngeal secretion mNGS on March 11 (Figure 1c). However, there was no significant improvement after active anti-infective treatment and the patient died 45 days after admission (Supplementary Figure 3 and Supplementary Table 2).

**FIGURE 2:**
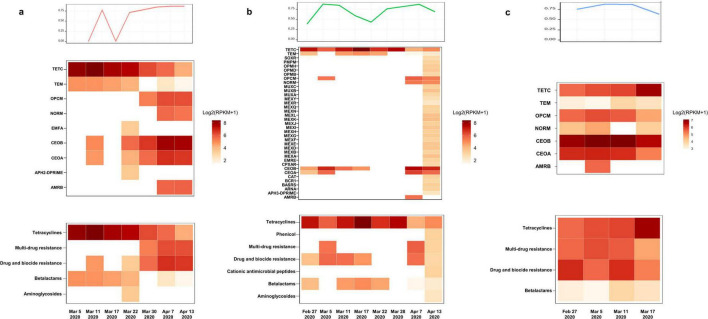
Antibiotic-resistant genes (ARGs) detection through metagenomic sequencing workflow. The levels of *CEOA*, *CEOB*, and *OPCM* were consistent with the trends in the relative abundance of *B. multivorans*. The related proteins expressed by these genes constitute the CeoAB-OpcM efflux pump, which may cause resistance to fluoroquinolones such as levofloxacin and aminoglycosides such as amikacin. **(a)** A total of 9 antibiotic-resistant genes (ARGs) were detected in 7 pharyngeal secretion samples collected from patient 1 at different time points through metagenomic sequencing workflow; **(b)** a total of 37 ARGs were detected in 8 pharyngeal secretion samples collected from patient 2; **(c)** a total of 7 ARGs were detected in 4 pharyngeal secretion samples collected from patient 3.

According to the results of the antimicrobial susceptibility testing of clinically isolated *B. cepacia* and literature reports, the three patients successively treated with sulfamethoxazole Co., ceftazidime, ceftazidime-avibactam, levofloxacin, and other first-line treatment for *B. multivorans* infection (Figure 1). The relative abundance of *B. multivorans* with a similar trend were observed, indicating the possible development of drug resistance. Two of the three patients died during hospitalization despite modification of antimicrobial therapy accordingly to the results of mNGS and drug sensitivity tests. They responded poorly to antimicrobial treatment. Therefore, a retrospective analysis of drug resistance in these patients was performed through the mNGS workflow. A total of 40 ARGs were detected, including *CEOA*, *CEOB*, *OPCM*, *TETC*, *TEM*, *NORM*, *EFMA*, and *AMRB* (Figure 2). Significantly, the levels of *CEOA*, *CEOB*, and *OPCM* were consistent with the trends in the relative abundance of *B. multivorans*. *CEOA* and *CEOB* were related to drug and biocide resistance, which contains a variety of antibiotic efflux pumps. Moreover, *OPCM* was associated with multi-drug resistance that resistant to two or more different antibiotics. Resistance nodulation cell division drug efflux pump CeoAB-OpcM has been reported to cause resistance to fluoroquinolones and aminoglycosides (10).

**FIGURE 3:**
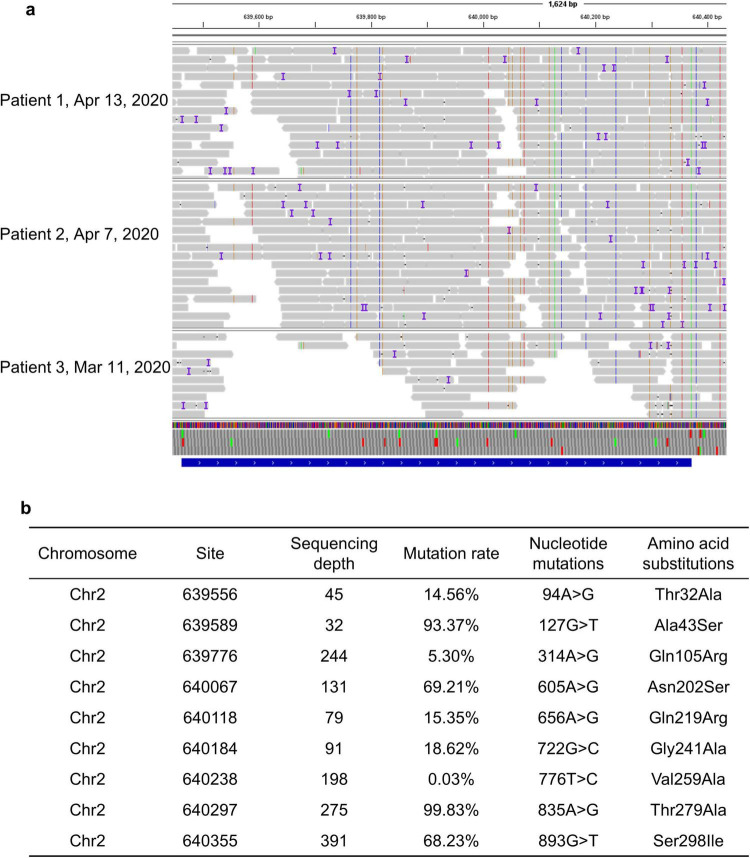
**(a)** Single nucleotide polymorphism analysis of PenA through SAMtools; **(b)** nine novel non-synonymous mutations in PenA of *B. multivorans*.

Single nucleotide polymorphism (SNP) analysis of *PenA* through SAMtools showed that the SNP sites of *PenA* in pharyngeal secretion samples collected from divergent patient at different time points were consistent, prompting that *B. multivorans* probably originated from the same strain and no new mutations emerged during the treatment period (Figure 3a). Most notably, we identified nine previously uncharacterized non-synonymous mutations in *PenA*, which codes for a class A β-lactamase and was involved in resistance to β-lactam antibiotics. These mutations lead to amino acid changes Thr32Ala, Ala43Ser, Gln105Arg, Asn202Ser, Gln219Arg, Gly241Ala, Val259Ala, Thr279Ala, and Ser298Ile compared to *B. multivorans* ATCC 17616, that may associate with the decreased sensitivity of *B. multivorans* to ceftazidime and ceftazidime-avibactam (Figure 3b).

## Discussion

*Burkholderia multivorans*, a Gram-negative bacterium, is a common etiologic agent causing nosocomial infection and diseases in immunocompromised individuals and cystic fibrosis sufferers (11, 12). A prospective analysis indicated that *B. multivorans* infection was strongly associated with adverse clinical outcomes. Hanulik et al. indicated that of the 46 patients isolated with *B. multivorans*, 23.9% cases died, of which 8 deaths were attribute to hospital-acquired pneumonia (13). The present study highlights the extreme complexity of *B. multivorans* infection in patients with COVID-19 in the intensive care unit. However, the interaction between SARS-CoV-2 and *B. multivorans* infection is poorly understood. Within the first few days after SARS-CoV-2 infection, critically ill patient with COVID-19 generally develop distorted respiratory tract or an imbalance in the lung flora, which can further progress into secondary bacterial or fungal infection few weeks later (14). Moreover, patients infected by *B. multivorans* usually exhibit a severe systemic inflammatory response, resulting in deterioration of the condition and triggering critical acute respiratory distress syndrome (2).

Antimicrobial resistance poses a significant threat to global health and is projected to cause 10 million deaths each year by 2050 (15). The resistance of *B. multivorans* to β -lactam is mainly driven by the generation of β-lactamases, which can hydrolyze β-lactam and prevent them from reaching the targets of penicillin-binding proteins (3). *B. multivorans* possess two types of chromosomal β-lactamases, PenA and a class C β-lactamases (AmpC). The PenA1 β -lactamase from *B. multivorans* ATCC 17616 is responsible for extending the spectrum of β -lactam resistance, while AmpC1 from *B. multivorans* ATCC 17616 has a very narrow spectrum and contributes very little to β -lactam resistance (16). Besides, PenA enzyme is similar to KPC-2, the most clinically important serine carbapenemase (17). Infections caused by *B. multivorans* is challenging to treat due to its ability to evade the action of multiple antimicrobial agents through intrinsic and acquired resistance mechanisms. On one hand, BCC microorganisms are intrinsically resistant to a variety of antibiotics, including β-lactams, fluoroquinolones, aminoglycosides, and polymyxins (18). Additionally, efflux pumps are crucial players in *B. multivorans* drug resistance. The synergistic effect between efflux and reduced outer membrane permeability was recognized as a common theme in the increased resistance exhibited by non-fermentative Gram-negative bacteria such as BCC (10). An early report indicated an outer membrane lipoprotein, OpcM, was implicated in resistance to several antibiotics (ciprofloxacin, trimethoprim, and chloramphenicol) (19). Notably, OpcM is the outer membrane channel of the efflux pump of the resistance nodulation cell division family. In the present study, we observed a higher abundance of resistance genes, among which *CEOA*, *CEOB*, and *OPCM* were associated with BCC, which was consistent with the trends in the relative abundance of *B. multivorans*. The related proteins expressed by these genes constitute the CeoAB-OpcM efflux pump, lead to resistance to fluoroquinolones such as levofloxacin and aminoglycosides such as amikacin (10). Moreover, *B. multivorans* is resistant to polymyxins. The *hpnN* gene of *B. multivorans* encodes the integrated membrane protein of the hpnN transporter family, which is responsible for shuttling hopanoids to the outer membrane (18). Hopanoid biosynthesis is involved in regulating outer membrane stability and permeability, contributing to polymyxin resistance (20). On the other, drug target alternations are key players of drug resistance. The Ω-loop of *PenA* emerges as a “hot spot” for obtaining single amino acid substitution that extends the substrate heterogeneity of *PenA* in clinically isolated *B. multivorans* (3). Substitutions at residues 164, 167, 169, and 179 of *PenA* were often observed in clinical isolates, conferring resistance to ceftazidime (5).

Although it has been recognized that *B. multivorans* infection is associated with an accelerated decline in respiratory function and increased morbidity and mortality, there is a lack of evidence-based antibiotic therapy for this infection. Ceftazidime and trimethoprim-sulfamethoxazole are recommended as the preferred treatment for infections caused by *B. multivorans*. Unfortunately, resistance to ceftazidime is increasing and can develop rapidly during the treatment, that poses a major threat to populations vulnerable to *B. multivorans* infection (5). Here, we identified nine previously uncharacterized non-synonymous mutations in *PenA*. These mutations lead to amino acid changes Thr32Ala, Ala43Ser, Gln105Arg, Asn202Ser, Gln219Arg, Gly241Ala, Val259Ala, Thr279Ala, and Ser298Ile, that may associate with the decreased sensitivity of *B. multivorans* to ceftazidime and ceftazidime-avibactam. As monotherapy may occur drug resistance, some experts would consider initial treatment with multiple antibiotics followed by a combination of two or three drugs depending on the drug susceptibility results. *B. multivorans* infection might require prolonged antibiotic courses and need to be closely monitored. In addition, bacteriophage (phage) therapy is a promising and potential alternative treatment for *Burkholderia* infections (21, 22). However, three documented cases of phage therapy for Burkholderia infection were unsuccessful and all three patients died (23–25). The prophages existing in the genomes of the *Burkholderia spp.* are potential useful starting points for the isolation and development of novel phages for phage therapy (21).

In the present study, we applied mNGS to pharyngeal secretion and the results showed *B. multivorans*. Subsequent conventional microbiological examination confirmed these results. Generally, mNGS allows for the direct detection of a wide range of pathogens. The wide detection spectrum, fast, and high sensitivity of mNGS are critical in the management of severe infections, where timely diagnosis can significantly impact patient outcomes (26). Generally, mNGS has the potential to sequence antibiotic resistance genes, providing valuable information on resistance mechanisms. This capability is essential to guide treatment regimen and improve antibiotic stewardship by helping to select the most effective antibiotics and preventing the spread of resistance. Moreover, the technology is transforming the landscape of clinical microbiology laboratories by providing a more comprehensive view of the microbial landscape in infections, which can lead to more accurate diagnoses and better patient management. Overall, mNGS is a powerful microbial detection tool that provides a comprehensive, sensitive, and rapid method for pathogen detection and drug resistance analysis, which is critical for improving diagnostic accuracy and guiding clinical decision-making in infectious disease management.

## Conclusion

In conclusion, this case series describes the respiratory microbiome alternations and antibiotic-resistant genes during the treatment of *B. multivorans* infection through mNGS. Additionally, we reported nine previously uncharacterized non-synonymous mutations in PenA of *B. multivorans*. These mutations lead to amino acid changes Thr32Ala, Ala43Ser, Gln105Arg, Asn202Ser, Gln219Arg, Gly241Ala, Val259Ala, Thr279Ala, and Ser298Ile that may associate with resistance to β-lactam antibiotics. As monotherapy may occur drug resistance, *B. multivorans* infection should be initially treated with multiple antibiotics, followed by a combination of two or three drugs depending on the drug susceptibility results. Moreover, *B. multivorans* infection might require prolonged antibiotic courses and need to be closely monitored. Moreover, for critically ill patients or immunocompromised individuals facing difficult or complex infectious diseases, it is recommended to use mNGS to optimize antibiotic treatment strategies, thereby improving therapeutic outcomes and reducing the unnecessary use of antibiotics.

## Data Availability

The raw data supporting the conclusions of this article will be made available by the authors, without undue reservation.
