# Supplementary material for: Pulmonary microbial spectrum of Burkholderia multivorans infection identified by metagenomic sequencing
Source: Front Med (Lausanne). 2025 Jun 17;12:1577363. doi: 10.3389/fmed.2025.1577363 (PMC12209342; doi:10.3389/fmed.2025.1577363)
Supplement: Supplementary file 1 [file Table_1.docx]

**Table S1. The schedule of pharyngeal secretion samples collection.**

| Cases | Sample collection time point |
| --- | --- |
| Patient 1 | Mar 5, 2020 |
|  | Mar 11, 2020 |
|  | Mar 17, 2020 |
|  | Mar 22, 2020 |
|  | Mar 30, 2020 |
|  | Apr 7, 2020 |
|  | Apr 13, 2020 |
| Patient 2 | Feb 27, 2020 |
|  | Mar 5, 2020 |
|  | Mar 11, 2020 |
|  | Mar 17, 2020 |
|  | Mar 22, 2020 |
|  | Mar 28, 2020 |
|  | Apr 7, 2020 |
|  | Apr 13, 2020 |
| Patient 3 | Feb 27, 2020 |
|  | Mar 5, 2020 |
|  | Mar 11, 2020 |
|  | Mar 17, 2020 |

**Table S2. Antibiotic medication during hospitalization.**

| Cases | Date | Antibiotic medication |
| --- | --- | --- |
| Patient1 | 2020.2.10-2020.2.22 | Meropenem (1g ivgtt once every 8h) |
|  | 2020.2.15-2020.2.22 | Vancomycin (1000mg ivgtt once every 12h) |
|  | 2020.2.19-2020.3.18 | Caspofungin (50mg ivgtt once a day) |
|  | 2020.2.22-2020.2.28 | Cefperazone-Sulbactam (3g ivgtt once every 8h) |
|  | 2020.2.22-2020.2.25 | Tigecycline (100mg ivgtt once every 12h) |
|  | 2020.2.25-2020.2.28 | Moxifloxacin (250mg ivgtt once a day) |
|  | 2020.2.28-2020.3.11 | Compound sulfamethoxazole (2 pills once every 8h) |
|  | 2020.2.28-2020.3.13 | Ceftazidime-avibactam (2.5g ivgtt once every 8h) |
|  | 2020.3.11-2020.3.19 | Amikacin (aerosol inhalation once every 12h) |
|  | 2020.3.13-2020.3.17 | Ceftazidime (2g ivgtt once every 8h) |
|  | 2020.3.14-2020.3.20 | Levofloxacin (once a day) |
|  | 2020.3.17-2020.3.22 | Ceftazidime (1g ivgtt once every 12h) |
|  | 2020.3.31-2020.4.4 | Meropenem (1g ivgtt once every 8h) |
|  | 2020.4.4-2020.4.6 | Ceftazidime-avibactam (2.5g ivgtt once every 8h) |
|  | 2020.4.5-2020.4.23 | Caspofungin (50mg ivgtt once a day) |
|  | 2020.4.5-2020.4.16 | Levofloxacin (once a day) |
|  | 2020.4.7-2020.4.23 | Ceftazidime-avibactam (2.5g ivgtt once every 12h) |
|  | 2020.5.6-2020.5.7 | Cefperazone-Sulbactam (3g ivgtt once every 8h) |
|  | 2020.5.13-2020.5.18 | Cefixime (100mg once every 12h) |
| Patient 2 | 2020.2.6-2020.2.9 | Moxifloxacin (once a day) |
|  | 2020.2.10-2020.2.16 | Biapenem (once every 12h) |
|  | 2020.2.16-2020.3.2 | Caspofungin (50mg ivgtt once a day) |
|  | 2020.2.16-2020.2.22 | Meropenem (1g ivgtt once every 8h) |
|  | 2020.2.16-2020.2.21 | Vancomycin (1000mg ivgtt once every 12h) |
|  | 2020.2.22-2020.3.3 | Cefperazone-Sulbactam (3g ivgtt once every 8h) |
|  | 2020.3.2-2020.3.6 | Caspofungin (50mg ivgtt once a day) |
|  | 2020.3.3-2020.3.8 | Ceftazidime-avibactam (2.5g ivgtt once every 8h) |
|  | 2020.3.8-2020.3.9 | Ceftazidime-avibactam (2.5g ivgtt once every 12h) |
|  | 2020.3.9-2020.3.26 | Amikacin (0.2g aerosol inhalation once every 12h) |
|  | 2020.3.9-2020.3.13 | Compound sulfamethoxazole (2 pills once every 6h) |
|  | 2020.3.12-2020.3.24 | Vancomycin (1000mg ivgtt once every 12h) |
|  | 2020.3.13-2020.3.24 | Ceftazidime (2g ivgtt once every 8h) |
|  | 2020.4.12-2020.4.22 | Ceftazidime-avibactam (2.5g ivgtt once every 8h) |
|  | 2020.4.13-2020.4.24 | Vancomycin (1000mg ivgtt once every 12h) |
|  | 2020.4.15-2020.4.22 | Caspofungin (50mg ivgtt once a day) |
|  | 2020.4.22-2020.4.24 | Meropenem (1g ivgtt once every 8h) |
| Patient 3 | 2020.2.8-2020.2.19 | Biapenem (once every 12h) |
|  | 2020.2.14-2020.2.23 | Vancomycin (1000mg ivgtt once every 12h) |
|  | 2020.2.19-2020.2.25 | Meropenem (1g ivgtt once every 8h) |
|  | 2020.2.19-2020.2.21 | Caspofungin (50mg ivgtt once a day) |
|  | 2020.2.22-2020.2.25 | Tigecycline (100mg ivgtt once every 12h) |
|  | 2020.2.25-2020.2.28 | Cefperazone-Sulbactam (3g ivgtt once every 8h) |
|  | 2020.2.28-2020.3.8 | Piperacillin-tazobactam (4.5g ivgtt once every 8h) |
|  | 2020.3.1-2020.3.16 | Caspofungin (50mg ivgtt once a day) |
|  | 2020.3.6-2020.3.11 | Ceftazidime-avibactam (2.5g ivgtt once every 8h) |
|  | 2020.3.12-2020.3.17 | Levofloxacin (once a day) |
|  | 2020.3.12-2020.3.17 | Ceftazidime (2g ivgtt once every 8h) |


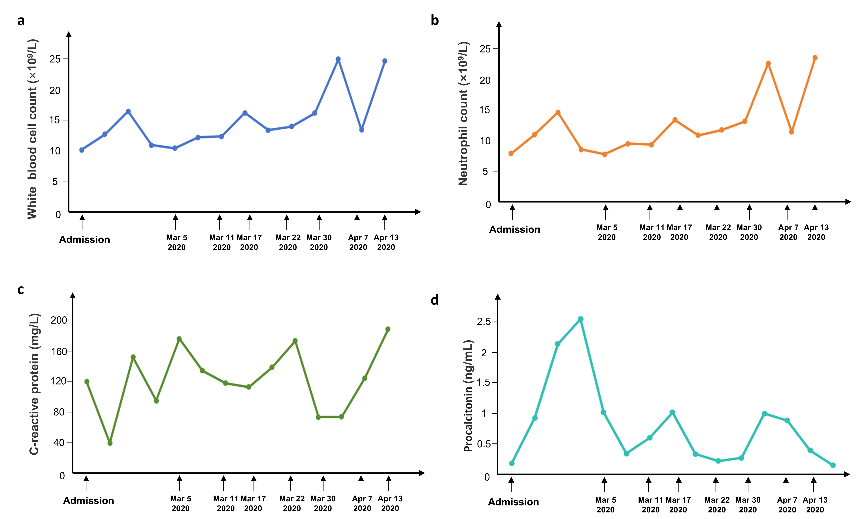


**Figure S1.** The changes in inflammatory markers and procalcitonin of patient 1 during hospitalization. (a) white blood cell count, (b) neutrophil count, (c) C-reactive protein, (d) procalcitonin.


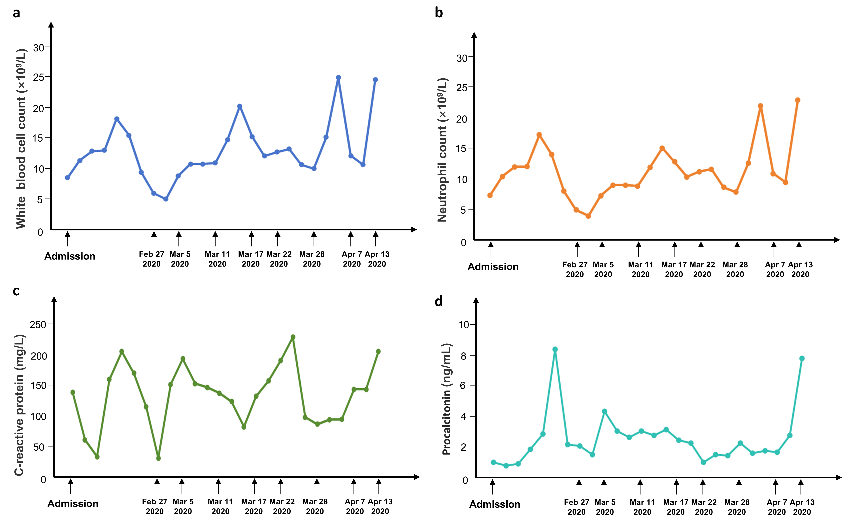


**Figure S2.** The changes in inflammatory markers and procalcitonin of patient 2 during hospitalization. (a) white blood cell count, (b) neutrophil count, (c) C-reactive protein, (d) procalcitonin.


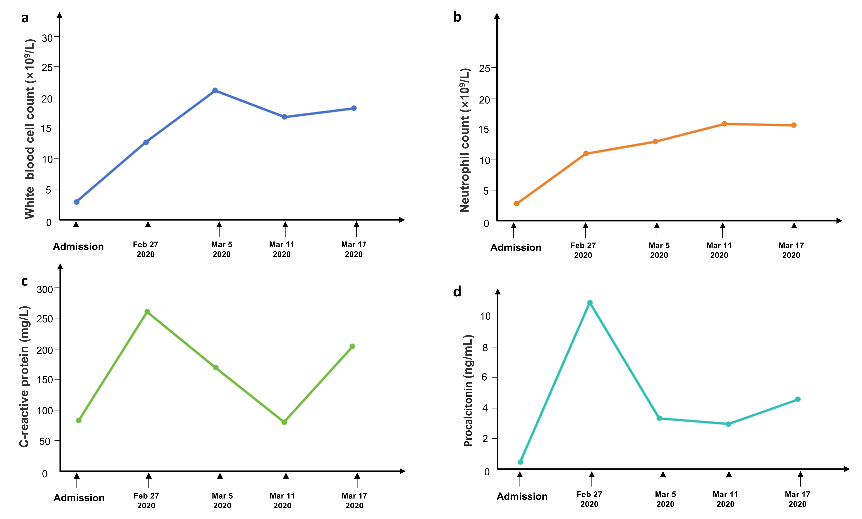


**Figure S3.** The changes in inflammatory markers and procalcitonin of patient 3 during hospitalization. (a) white blood cell count, (b) neutrophil count, (c) C-reactive protein, (d) procalcitonin.
